# Practice of Scoptzi in Roumania

**Published:** 1873-12

**Authors:** Robert Battey

**Affiliations:** Professor of Obstetrics in the Atlanta Medical College


ATLANTA

MEDICAL AND SURGICAL JOURNAL.

VOL. XI.] DECEMBER—1873. [NO. 9.

### Original Communications.

### PRACTICE OF SCOPTZI IN ROUMANIA.

BY ROBERT BATTEY, M.D.,

PROFESSOR OF OBSTETRICS IN THE ATLANTA MEDICAL COLLEGE.

In an address before the Georgia Medical Association, at its last session in Atlanta, upon the subject of Normal Ovariotomy, published in the April and May numbers of this Journal, of the current year—the writer used the following language: "Allusion is also made in *Appleton's Journal*, of September 21st, 1872, to the same operation, practiced among the Scoptz, a religious sect in Roumania, as a religious rite, as circumcission is among the Jews. It appears that a fatal case had aroused the authorities to take cognizance of their doings, and as they are represented to have long practiced this rite, it would seem that it had been attended, probably, with no great degree of fatality, or attention would have been earlier drawn to the subject."

A careful search of the sources of information within reach of the writer, including the older edition of the voluminous *Edinburg Encyclopœdia* in quarto, and the recent editions of *Appleton* and *Chambers*, has entirely failed to shed any light upon the subject. Indeed, the Dictionaries at hand do not even contain the word Scopt, or Scopet, or its congener.

Believing that valuable information might be elicited from the Scoptz themselves, which would possibly have an important bearing upon the operation in question, the writer took steps, at once, to procure such definite knowledge upon the subject as could be drawn from authentic and reliable sources.

Through the courtesy of the Hon. Hamilton Fish, Secretary of State, inquiry was instituted through the representative of our Government at Bucharest, in Roumania, at St. Petersburg, and at Moscow, in Russia.

From Benjamin F. Peixotto, Esq., Consul of the United States at Bucharest, Roumania, the following communication has been received, namely:

*Memorandum on the practice of Scoptzi or castration, by the males and females of the religious sect known in Roumania as Scopetii or Scopts.*

There is a religious sect in Roumania known as Scopetii or Scoptzi, and numbering, in all, in the provinces of Wallachia, Moldavia and Roumanian Bessarabia, five hundred and thirty-three persons. They originally came from Russia, where the largest number still continue to reside.

This sect must not be confounded with the Lipovans proper, whose population in Roumania is over fifteen thousand; as the Lipovans have no such practice as Scoptzi or castration in their creed.

*Castration of the Males.*—Scoptzi or castration, consisted, with the old Scopez, in cutting off the testicles, which they called the "twin members." It was done by cauterizing the petty sack with a red-hot iron; an operation known as the "baptism of fire." Later, the separation was performed by razors, knives or other sharp instruments, with which the *petit sac* was cut off, after it had previously been strongly tied with a string. Subsequently cauterization is sometimes employed as a means of stopping the flowing of blood.

But fanaticism did not rest here. According to the declaration of the Scopetz themselves, the absence of the testicles does not destroy altogether the bodily concupiscency, and those castrated in this way do not lose the faculty of cohabitation, although the act is performed without the ejection of sperma, and is effected with great effort and extreme fatigue. To arrive, then, at perfect chastity, and the utter extinction of passion, the fanatics decided to remove even the member (penis) which is called *cheia abyzulin* (clince bezduy) the "*key of the abyss.*" This operation which sometimes takes place several years after the removal of the testicles, and sometimes together with the first operation is called *botezu deplinu*, *i. e.*, the perfect baptism, or *pecetei imperatexi*, *i. e.*, the "imperial seal"; and is effected with an axe or hatchet. This operation appears to be of more recent date, and was first introduced about the year 1816. This produced a scism in the sect, whose vestiges are still existing. The old Scopez who followed the first operation only, consider the "imperial seal" as a criminal innovation originated with the Scopetz across the Moseon, whom they call "dogs."

Besides the principal forms of castration, there are also others. There is a sect called "Perevertysii" or Twisters—principally in the province of Tamboonlu. These do not cut off the members, but from childhood twist the *funiculi spermatici*, and thus stop every organic communication between them and the body, which prevents the formation of the sperma within them, and produces the same effect as castration. In 1841–'42 another sect was discovered, founded by a peasant—Kutkin, who is suspected to castrate by splitting or piercing the *funiculi*, which has the same result as the twisting of the Perevertysii. Physicians believe this to be a very difficult operation, as the cutting of the veins must produce flowing of blood to the imminent peril of life. If successfully done, the castration cannot be recognized.

### CASTRATION OF WOMEN.

Not men only are submitted to this operation, but also women, who take the name of "*Scopcichi.*" The operation with women applies to the breasts and to the genitals. Sometimes their breasts are entirely cut off; sometimes only the nipples are cut, burned or corroded; sometimes they cut out only the glands from under the breasts, especially from under the left breast.

At the genital parts they cut the clytoris, the labia minor, and sometimes the labia major. Such mutilations, however, do not in reality produce the same effect as the removal of the testicles of the male. *The real castration with women could be effected only by the removal of the ovaries*, but this operation is considered by modern physicians, if not altogether impossible, at least dubious.

Learned medical men, however, affirm that the cutting of both breasts is almost equal to real castration, for the breasts being in close connection with the womb, their absence must deprive women of the faculty of conception and concupiscency at the time of cohabitation.

This is said to be confirmed by the fact that the so mutilated women are commonly distinguished in their outward appearance by the same deformity, faded complexion, and want of elasticity and spirit, in the very bloom of their lives, as with the male castrated. All other mutilations of women are not real castrations if they leave them the faculty of cohabitation and pregnancy.

Generally the mutilated women have a yellow, wrinkled complexion, small breasts, etc. This cannot be explained by their abstinence; there have been observed cases of great corruption of these women; none of them however gave birth to children. It is, therefore, to be suspected that they cohabit with the Scopetz who have not the "imperial seal," and from this unnatural and unsatisfied irritation springs their state of weakness and infirmity.

Old Scoptzi affirm that the castration of women is a novelty of Moscow, introduced at St. Petersburg at the time of the second "seal" for men, in 1816. Budilin asserts that the castration of women has two degrees; the first being the injury of the womb, and the cutting of the clytoris; the second, the removal of the breasts, which is done by instruments having the shape of a knife and fork.

In Bucharest there are two hundred Scopetz, who are principally engaged in driving public vehicles which they own. They appear to love horses, having the best and swiftest, and driving like demons. They have also a passion for hoarding money. They are all well-to-do. They are of a pale yellow complexion and grave-like visages. A short time after their castration, their beards fall out and their voices change to the thinnest feminine key. They are all of Russian birth or extraction, and as their number die out, they appear to import others in their place.

I have not yet been successful in discovering whether they now castrate their women; they will not speak upon the subject for love or money. In Jassy, however, there was recently a case before the courts, prosecuting some of the sect, for cutting off the breasts of a young woman, whom it was claimed, they had converted. Before they are castrated they are permitted to marry and have one child; then they are worked upon by the fervor of religious zeal until the act is performed."

---

Proposed Monument to Eustachius.—The city of Sanseverino-Marche, Italy, has resolved to erect a monument to its greatest citizen, Bartholomew Eustachius, the great anatomist, philosopher and physician of the sixteenth century. Subscriptions will be received at the office of the Annali Universali di Medicina, Milan.—*New York Medical Journal.*

